# Intersectionality in the lives of people with intellectual disability and their caregivers: insights from a study on respite care in the South African context

**DOI:** 10.3389/fpsyt.2026.1844351

**Published:** 2026-07-03

**Authors:** Toni Abrahams, Sharon Kleintjes

**Affiliations:** 1 Department of Psychiatry and Mental Health, Faculty of Health Sciences, University of Cape Town, Cape Town, South Africa; 2 Department of Health and Wellness, Western Cape Government, Cape Town, South Africa

**Keywords:** intellectual disability, intersectionality, policy, practice, respite care, service, South Africa, support

## Abstract

**Background:**

This article advocates for an explicit intersectional lens when conducting research with people with intellectual disability (ID) in South Africa (SA). An intersectional lens spotlights how identity categories, power relations and structures, structural inequities, and disability converge to heighten disadvantage and marginalisation.

**Methods:**

This paper engages in a secondary analysis of a doctoral thesis on respite care for persons with ID in SA, a mixed-method, inclusive study entailing interviews, a policy analysis, and a national survey where the integrated data informed service considerations for this disability support service. For this paper, the thesis itself was re-analysed using framework analysis informed by intersectional theory. In doing so, the outputs were reconfigured to expose the intersections of privilege and marginalisation hiding within the broader narrative.

**Findings:**

Intersectionality reveals how people with ID and their caregivers in SA experience barriers to accessing quality respite care, affecting health and wellbeing. The lived experience of poor, black people with ID and, especially, their female caregivers is shaped by persisting racial disadvantage arising from interconnected structural inequities, including socio-economic inequality, geographic disparities, gendered care responsibilities, ableism, disablism, disability hierarchies, and cultural concerns, producing layered disadvantage in accessing responsive services. Intersectionality highlights the importance of focussing on the experience of people with ID who encounter particular marginalisations and disadvantage related to their disability. These insights inform recommendations to improve respite care for people with ID and their families.

**Implications:**

The paper illustrates the value of this lens, for which intersectional methodologies are needed and can be harnessed in future ID research in SA.

## 1 Introduction

The concept of intersectionality foregrounds how various aspects of social identity, such as race, socio-economic status, gender, and disability, can interact to co-constitute disadvantage and exclusion, producing specific and compounded forms of marginalisation in a group’s lived experience (1–4). People with intellectual disability (ID) are amongst the more marginalised people with disability, experiencing rights infringements, in particular in health, education, employment, and reduced protection from poverty (5–7). By considering diverse and contextually influenced experiences of disability (3, 4, 8), intersectionality can enrich research, as it provides a critical framework for understanding how people with ID and their family caregivers experience multiple and overlapping forms of inequality. It does so through its attention to power systems and relations such as racism, patriarchy, disablism, and ableism, which shape their lives (1). ID research in South Africa (SA) often implicitly contains data on overlapping systems of marginalisation produced through apartheid legacies, poverty, race, gender, and disability, for example, Mkabile and Swartz (9) and Tyabashe-Phume and Kleintjes (10). This paper advocates for the importance of an explicit intersectional lens when conducting research regarding people with ID in SA by illuminating how experiences of respite care (RC) are shaped by intersecting structural inequities and identity categories. It retrospectively re-analyses doctoral findings on respite care as a case example where intersectionality was not originally used as an explicit analytic lens, but deepened and enriched understanding of the research focus. Respite care is a disability support service that provides temporary breaks from caregiving for caregivers, families, and care recipients with ID. Quality RC can potentially enhance the health and wellbeing of this population, especially in low- and middle-income countries where families frequently assume lifelong caregiving responsibilities in the context of inadequate services (7, 11, 12).

## 2 Methods

The inclusive, mixed-method doctoral study included four sub-studies: a scoping review, an RC-related South African policy analysis, a national RC service survey, and 51 semi-structured interviews conducted with people with ID (PWID), family caregivers (CGs), service providers (SPs), and key stakeholders (KSs) in the health, social, and disability sectors. The study analysed the data through health systems dynamic (13), respite (14), and rights-based lenses (15) to develop a set of RC service considerations for the public sector in SA, as detailed in Abrahams and Kleintjes (16). The study used policy and framework analyses for the RC policy review and interviews, respectively, while descriptive analyses were used for the scoping review and survey. These data were integrated using joint displays and represented in the synthesised body of data within the study thesis. For this paper, returning to the full and large data set of the study was not feasible; thus, the thesis itself was re-analysed. This secondary analysis drew on the work of intersectional theorists (1, 2, 4), using a five-step framework analysis approach (17). An initial coding frame of *a priori* themes based on intersectional theories was developed. Data in the thesis were then indexed using the coding frame and based on the author’s deep familiarity with the data in the doctoral study. The data were charted into an Excel-based data extraction matrix. Original data sources were consulted to ensure that charted data remained grounded in the original themes within the data and to identify relevant quotations or findings to illustrate intersectional trends. Mapping and interpretation comprised a descriptive synthesis of themes, subthemes, and their implications for the study’s focus.

## 3 Intersectional insights related to respite care for people with intellectual disability

Below, we elaborate on how people with ID and their families in SA are affected by intersecting and compounded inequalities, deepening the marginalisation which disability already brings to their lived experience.

*Spatial-economic inequalities* identified in the analytic process centred on the historical and intergenerational intersection of race, geographical location, and socio-economic disadvantage. In many instances, respondents’ views were underpinned by the impact of pre-apartheid and apartheid legislation, which restricted black South Africans’ land ownership, movement, and residence, confining many to underdeveloped rural homelands and poor urban townships, still impacting black poverty today. The experience of RC access is situated within this racialised history.

‘*If I was White, I was going to be free because white people leave their children with their nannies day and night. So I’m black, I do not have enough money to pay someone to look after my child at night.*’ *(CG, 3)*

Some caregivers living in informal housing in black townships were interested in in-home RC (respite care in the family home), which service providers could not easily offer due to space, infrastructure, safety, and regulatory constraints. In peri-urban settings, caregivers particularly struggled to access services due to expensive and inaccessible transport and long travel distances to areas where RC services were located. The survey found that only two out of the 17 surveyed RC services offered free services needed by poor families, but could not prioritise this expense due to their socio-economic position.

‘*…we use his grant for his nappies and his food, clothes and toiletries … but now, because there are so many people in the house … it’s not enough because I’m not working*’ *(CG, 3)*

Lack of RC access compounded poverty because employment opportunities could not be sought due to inadequate care support to enable work.

‘*I can’t find a job that makes me leave late enough to be home with her and come home early enough and be home. I’m literally not able to leave because she gets home different times and she can’t be home alone.*’ *(CG, 5)*

Service providers in poorer communities, in turn, reported struggling to meet state regulatory and licensing requirements needed to access state funding, which would permit low or no service fees. This compounded the poor availability of quality RC services.

Black caregivers and people with ID also spoke of the challenges experienced by young people with ID in accessing education to enable adult access to post-school employment, fuelling a lack of secondary respite (a break from caregiving resulting from the provision of another service not primarily intended to provide respite) for both caregivers and persons with ID. One noted:

‘*if you go there [Department of education], you won’t find school for your child. You must go there about 1000 times … to get [a school].*’ *(CG, 10)*

The findings showed how geographical location intersects with historically racialised spatial economic underdevelopment under apartheid, producing and maintaining layered disadvantage in accessing RC services. The survey, for example, found that 82% of services were in the Western Cape and Gauteng provinces, two main apartheid-era and current investment and development hubs.

Interwoven with the above, structural inequities in service provision intersect with gendered care. Women’s roles in caregiving were influenced by both patriarchal expectations and cultural attributions about care responsibilities. Historical patterns of male migrant labour required by pass laws, which contributed to entrenching caregiving roles for women left in the homelands to support families, continued to impact female caregivers. The role of fathers in fathering and caregiving was reported as still a struggle, with fathers often described as uninvolved, leaving mothers to shoulder the care burden.

‘*He [PWID’s father] made it clear in court that he wants nothing to do with her. The only time he said he should be involved is when she passes away.*’ *(CG, 13)*

Stigma associated with ID and disability, and patriarchal relationality impacted fathers who were more likely to renege on care responsibilities, leaving full-time care to female caregivers.

‘*[PWID Name] was not born like this … he became like this [disabled] … That is why I think his father left because of his condition. We never fought, there was no quarrel between us. He packed his bags and he left.*’ *(CG, 20)*

Women (mothers, grandmothers, aunts, and sisters) in the study tended to assume primary caregiving roles, with those living in poverty or rural spaces experiencing caregiving as particularly difficult. They described emotional, physical, and economic strains associated with sustained care responsibilities in the context of limited informal (unpaid support usually from family and friends) and formal (paid or formalised support usually from public and private services) support.

‘*It is like looking after a 3-month baby for four years, you can imagine it is exhausting.*’ *(CG, 2)*

Female caregivers’ care responsibilities were further complicated by *comorbidity*, *disability severity*, and *lifespan issues*, which deepened their disadvantage in accessing supports. For example, caregivers of relatives with high support needs, often with other physical comorbid conditions, experienced particular challenges in accessing RC. This was also so for families of people with ID and behaviours that challenge (BTC) or psychiatric conditions.

‘*Then she said, oh, but they won’t let [PWID Name] stay at the [RC organisation] with her behaviour. I said, but now help me with the behaviour … And that never happened. We never met. I’ve never had a session…*’ *(CG, 20)*

Expenses linked to high support needs and incidental costs required for providing care in the home were not accounted for in limited disability-related grants.

‘*Just her nappies was R700 a month. That’s not her food, that’s not her schooling, that’s not her transport, that’s not her clothes … that’s not her medication.*’ *(CG, 22)*

At the same time, grants meant for their relative with ID often supported the entire family and were not and could not be prioritised for paying for RC.

‘*I went twice and now I’m supposed to go again, but then my mommy said it’s too expensive to go. (PWID, 3)’*

Geographical disparities clustered with this, significantly exacerbating accessibility problems for persons with multiple disabilities. A mother with a child with profound ID and physical disabilities living in an informal settlement shared:

‘*Taking her to those places [RC], having to take a bus … that is a lot … sometimes I must ask people for transport … she is heavy, and this chair cannot get into a bus. So now I must put her on my back … If they can only pick us up and drop us off because some taxi drivers, would even charge me more…*’ *(CG, 11)*

Given the interdependence of caregivers and care recipients with disability across the lifespan, *age-related difficulties* are also crucial considerations. Legislated service provisions for 18- to 59-year-old adults with ID, for example, are lacking in SA compared to children and older persons with ID, also impacting RC access for this age group.

‘*They go to school, a special school, they turn 18 and then there’s no service…. We’ve had OT, physio, speech therapy, we’ve had interventions from Social Development.… But then as soon as the child turns 18 and is classified as an adult, they are on their own recognisance.*’ *(KS, 7)*

Age also impacted family caregivers anxious about their care recipient’s future care in their impoverished communities. The burden on older caregivers was pronounced for those whose care recipients had complex behavioural support needs, which could see them excluded from RC access. Most often, older female caregivers were left to continue caregiving while experiencing declining health and reduced income and support, with less involved male family members finding the burden of care overwhelming when their support was eroded, as illustrated by this call from a father whose wife died:

‘*…you are my last call. If you can’t, I have to take his life and mine because I can’t leave him here and I can’t do this anymore.… I can’t even lift him anymore, I am too old … he’s getting aggressive and upset because I can’t do the things that he needs to do … Together we could have tried, but now I have nobody.*’ *(KS, 2)*

Woven through these dimensions are *ableism*, *hierarchies within disability*, *disablism*, *stigma*, *and cultural influences*, threaded through respondents’ accounts of intersecting racial, spatial, socio-economic, and gender disadvantage in living with ID. Ableism was evidenced in how disability was given lower priority in state departments.

‘*We took a budget cut of R11 million. That’s just for disability.… You would find that another programme would get a R10 million increase and then you get us.*’ *(KS, 6)*

Respondents also highlighted the tension between the value ascribed to people with ID versus those with other disabilities. For example, physical disabilities were seen to be prioritised in policy and funding.

‘*My biggest fight was everything was about physical disability.… Because everything is about wheelchairs.*’ *(SP, 3)*

In addition to ableism and differential treatment of ID, being a person with an *intellectual* disability created specific marginalisations. For example, infantilisation in RC services affected the dignity of adults with ID:

‘*She wants to be an adult so if they can put her in where adults are instead of putting her with the kids … [PWID] sees herself as an adult, she wants to help [rather] than being helped.*’ *(CG, 19)*

Assumptions of incapacity arising from ID also reduced RC service users with ID’s opportunities to exercise autonomy.

‘*If I want a cup of tea or a cup of coffee I make me here at home. Like by [RC name] you can’t make your [snack/tea], you must wait…*’ *(PWID, 2)*

Service providers reported that people with ID were often excluded from RC assessments due to communication impairments, instead focussing on professional and caregiver reports. The policy analysis and interviews showed that people with ID were excluded from general and RC-related policy development despite legislation encouraging inclusion. Exclusions such as these were, in part, linked to disability severity, where service providers lacked knowledge and skills to engage people with ID in a way that was responsive to their individual communication capacities.

Linked to disablism, experiences of stigma and discrimination imposed social isolation for respondents with ID and their caregivers, further marginalising them, impeding RC access and entrenching isolation:

‘*People … that don’t have a child like this and that doesn’t work in this field, they don’t understand. They reject you, they reject your child. You’re much in exile when you have a child like this…*’ *(CG, 6)*

The impact of psychoemotional disablism was evident in the shame, pathologisation, and blame experienced by caregivers and people with ID, leading to self-imposed isolation, resistance to RC utilisation, and resultant mental health problems in caregivers and their care-recipients.

‘*Most people ask why I am like this. And sometimes they would say silly things to me because of my situation. So I don’t go out of the yard to play with other kids.*’ *(PWID, 1)*

‘*Sometimes you feel like the body does not want to do anything. And the child is here, and you have no choice but to get up. Sometimes you don’t want to wake up at all. (CG, 18)*

RC utilisation was further impeded by disability stigma, where people with ID were hidden, reinforced by cultural attributions about ID aetiology and beliefs that discourage the use of such services, locking female caregivers in unsupported care responsibilities. Female caregivers embedded in collectivist traditions faced isolation due to service utilisation being seen as a rejection of caregiving responsibilities.

‘*…if you send your child to a home then you don’t want to take responsibility. You are neglecting your child…. It’s like you are putting all the work on someone else’s hand.*’ *(CG, 3)*

## 4 Discussion

An intersectional lens illuminated findings that show that people with ID and their families are disproportionately affected by interconnected structural inequities, including racialised socio-economic inequality, geographic disparities, historical disadvantage, gendered care responsibilities, disability hierarchies, and power relations of ablism, disablism, stigma, and culturally informed disability and care beliefs, which compound disability marginalisations. These inequities reflect persisting structural patterns of racialised resource distribution, which shape how families, in particular black families with relatives with ID, navigate support systems (18), highlighting what needs consideration in developing effective RC policies, service delivery, and funding streams (1).

South Africa’s post-apartheid constitution entrenches the right of all South Africans to a good life, with national policies supporting pro-poverty strategies, yet three decades into this new dispensation, apartheid’s legacy continues to disproportionately leave black people with ID and their families with intergenerational poverty and hardship despite the country’s status as an upper-middle-income country. An intersectional lens shows that this disadvantage extends deeply into their access to social- and health-related supports such as respite care. Legislation and policies are already in place, which prioritise addressing racial and socio-economic inequities through commitment to community-based disability services in SA’s underdeveloped, poverty-laden areas. These policies, however, are consistently poorly implemented across sectors, with the structural underpinnings of still-present social and economic disparities in SA preventing any significant changes within the public sector. The results of apartheid rural and urban planning are still in place, with mainly poor, black, and under-resourced communities lacking public health service provision, most significantly impacting people with disabilities (19). An intersectional lens elucidates how black people with ID and their caregivers are disproportionately affected by this. So too are they shown to have even fewer opportunities to extricate themselves from poverty due to reduced access to quality education and post-school employment opportunities, while caregivers struggle to access employment, competing against many others who do not have the lifelong burden of unsupported care. These disparities are deepened between those who live in urban versus rural spaces.

While there are these historically developed disparities, there is also a growing disquiet with the state’s inability to deliver on the structural conditions necessary to grow the economy. The current debates on state corruption and failure to deliver on state deliverables, pervasive and intergenerational poverty, high unemployment rates, and rising costs of living, violence, and crime all impact the lived experience of people with ID along with other citizens (20). Within this context, women, embedded in patriarchally informed gendered caregiving expectations (21), are multiply burdened by traditional cultural beliefs to stoically provide care (22) and render care in a structurally deficient context. This intersectional view supports the call for the implementation of national disability policies, provincial strategies, and service plans that should offer economic support, support boys and men to reimagine their roles, strengthen informal support networks, raise ID awareness, and provide targeted support beyond RC. RC service gaps cannot be adequately addressed without consideration of these broader issues. This aligns with intersectionally informed, equitable praxis for marginalised groups (1).

The findings also highlight the importance of casting an intersectional eye to understand the particularities of the lived experience of disability. RC for people with ID in SA provides one case example of how intersectional issues disproportionately affect segments of this already-marginalised population as result of ablism and how disadvantage experienced by people with disabilities is compounded for those with ID in this context. This disparity may be explained by the fact that people with ID face exclusion when general disability policies replace tailored support, causing unmet needs and resource competition (23). Disability policy should consider intersectional injustice, as institutional ableism disproportionately impacts those marginalised by race, class, gender, location, and mental distress (4). Legislative reform through an implemented Disability Act, fully domesticating the UN Convention on the Rights of Persons with Disabilities (2006), the activation of a twin-track approach to support mainstreaming and disability-specific services (24, 25), the explicit recognition of ID using disability-disaggregated definitions (15, 26, 27), and developing ID-specific RC-inclusive policy can address systemic ableism and inequalities identified through this lens. The findings further highlight how readily rights infringements occur for people with ID, compounded for those with more severe impairments (28). ID challenges intersectional frameworks that assume a stable disability identity, agency, and self-advocacy, overlooking varied recognition, intelligibility, and levels of agency amongst people with ID (4). This was evidenced in how a lack of communication supports can be mistaken for a lack of capacity, discouraging inclusion. The adoption of an ID-nuanced intersectional lens reveals the importance of advocacy for interdependent and inclusive practices (4), which, in the case of RC, can be brought to life through rights-based leadership, governance, and service delivery (29).

Issues related to disability severity, comorbidity, and age add complexity to the situation of people with ID in this context. The findings highlight how support needs can be differentially (un)met due to structural determinants and that disability severity is not rooted in biology alone, but is shaped by contexts of inequality (3). This was evident for people with ID and BTC who are at a higher risk for social exclusion but are excluded from essential support (30–33). An intersectional lens thus reinforces that people with ID with intensive and complex support needs should be prioritised for adequately funded provision (4) based on individual and family support needs. Furthermore, the challenges spotlighted for people with ID and caregivers in this context and at the intersection of age point to the need for legislative and policy improvements to adopt a lifespan approach, thereby ensuring that RC services better extend to adults with ID and provide preventative support for ageing caregivers. The latter is not simply beneficial, but urgent, to prevent the risk of care collapse as a result of compounded disadvantage for this cohort, which undermines the state’s obligation to deinstitutionalise people with ID (25).

Finally, this lens illuminates the particular challenges brought on when people with ID and their caregivers in this context also encounter disablism, disability stigma, and contributing cultural beliefs, which affect the uptake of services. Psycho-emotional disablism can cause emotional and relational harm (4), resulting in caregivers, already under strain due to their disadvantaged positions, counterintuitively being less likely to utilise much-needed RC. Differential access and utilisation, impacted by stigma and shame in communities, can have detrimental impacts on caregivers’ health and wellbeing and the rights of their care recipients (30, 34–36). Failing to account for variations in cultural and disability understandings may also worsen stigma for this population, negatively impacting the care experience (37). These intersectional findings highlight the need not only for culturally sensitive RC service delivery but also for addressing the broader power structures of disablism and disability stigma through awareness and advocacy.

## 5 Conclusion

Through the use of an intersectional lens, this paper demonstrates the deep and complex marginalisations that people with ID may experience, using RC as an example. By highlighting how disability is differentially experienced, it challenges homogeneous understandings of people’s experience of ID. It calls for rights-based and person- and family-centred RC policy and practice, treating support as political, not just a personal endeavour (2). A limitation of this paper is that it utilised framework analysis for a re-analysis of selected data from an original study that did not use an explicit intersectional lens. Greater depth in the analysis of the full dataset, beyond the scope of this paper, may have generated other findings of relevance to the study’s focus. The current paper, however, using this approach, generated very meaningful findings to inform RC policy and service directions and supports a call for more intersectionally informed ID research in SA, necessitating the development of rigorous intersectional analysis methods to inform research in this field.

## Data Availability

The original contributions presented in the paper are publicly available. This can be found here: http://hdl.handle.net/11427/42068.
